# Utilizing electronic health records to predict acute kidney injury risk and outcomes: workgroup statements from the 15^th^ ADQI Consensus Conference

**DOI:** 10.1186/s40697-016-0099-4

**Published:** 2016-02-26

**Authors:** Scott M. Sutherland, Lakhmir S. Chawla, Sandra L. Kane-Gill, Raymond K. Hsu, Andrew A. Kramer, Stuart L. Goldstein, John A. Kellum, Claudio Ronco, Sean M. Bagshaw, on behalf of the 15 ADQI Consensus Group

**Affiliations:** Division of Nephrology, Department of Pediatrics, Stanford University, 300 Pasteur Drive, Room G-306, Stanford, CA 94304 USA; Departments of Medicine and Critical Care, George Washington University Medical Center, Washington, DC USA; Departments of Pharmacy, Critical Care Medicine and Clinical Translational Sciences, University of Pittsburgh, Pittsburgh, PA USA; Department of Medicine, Division of Nephrology, University of California San Francisco, San Francisco, CA USA; Prescient Healthcare Consulting, LLC, Charlottesville, VA USA; Division of Pediatric Nephrology, Department of Pediatrics, Cincinnati Children’s Hospital Medical Center, Cincinnati, OH USA; Department of Critical Care Medicine, University of Pittsburgh, Pittsburgh, PA USA; Department of Nephrology, Dialysis and Transplantation, International Renal Research Institute of Vicenza, San Bortolo Hospital, Vicenza, Italy; Division of Critical Care, Faculty of Medicine and Dentistry, University of Alberta, Edmonton, Canada

**Keywords:** Acute kidney injury, Electronic health record, Electronic medical record, Prediction, Big data, Predictive analytics

## Abstract

The data contained within the electronic health record (EHR) is “big” from the standpoint of volume, velocity, and variety. These circumstances and the pervasive trend towards EHR adoption have sparked interest in applying big data predictive analytic techniques to EHR data. Acute kidney injury (AKI) is a condition well suited to prediction and risk forecasting; not only does the consensus definition for AKI allow temporal anchoring of events, but no treatments exist once AKI develops, underscoring the importance of early identification and prevention. The Acute Dialysis Quality Initiative (ADQI) convened a group of key opinion leaders and stakeholders to consider how best to approach AKI research and care in the “Big Data” era. This manuscript addresses the core elements of AKI risk prediction and outlines potential pathways and processes. We describe AKI prediction targets, feature selection, model development, and data display.

## Abrégé

Les données figurant dans les dossiers médicaux électroniques (DMÉ) sont considérables, tant au point de vue du volume que du débit ou de la variété. Ces trois caractéristiques et la tendance générale à adopter les DMÉ ont soulevé un intérêt pour appliquer les techniques d’analyse prédictive des mégadonnées aux données contenues dans les dossiers médicaux électroniques. L’insuffisance rénale aiguë (IRA) est une maladie qui convient parfaitement à une méthode de prévision et de prévention des risques: non seulement la définition acceptée de cette affection permet-elle un ancrage temporel des événements ; mais il n’existe aucun traitement une fois que la maladie est déclarée, ce qui montre l’importance d’une détection précoce. L’*Acute Dialysis Quality Initiative* (ADQI) a convoqué un groupe de travail constitué de leaders d’opinion et autres intervenants du milieu pour se pencher sur la meilleure façon d’approcher la recherche et les soins offerts aux patients atteints d’IRA en cette ère de mégadonnées. Le présent article traite des éléments centraux de la prévention des risques et en expose les procédures potentielles. Nous y décrivons les cibles de prévention de l’IRA, la sélection des paramètres, l’élaboration des modèles et l’affichage des données.

## Background

The term “big data” has traditionally been used to describe extraordinarily large and complex datasets. For many medical practitioners, this concept was initially epitomized by genomics – the colossal amount of discrete data generated by high throughput sequencing techniques required analytic methods ranging far beyond standard statistical approaches [1]. However, “omics” are now ubiquitous and “big data” has become vernacular in medicine [2, 3]. Clinical researchers are beginning to employ innovative, high-content analytic techniques capable of integrating and exploring the exceedingly large and diverse datasets contained within the electronic health record (EHR).

**Fig. 1:**
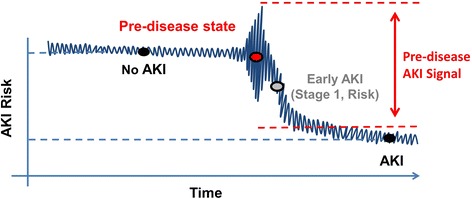
Signal Identification for AKI Development and Progression. Current consensus AKI definitions allow AKI events to be precisely anchored from a temporal standpoint, clearly defining a pre-disease state. As the patient progresses from “No AKI” to “AKI,” the pattern of data generated within the EHR changes, creating an “AKI signal” which can be identified through advanced analytic techniques. This signal can be translated into a prediction model which is capable of identifying patients at high risk for AKI development. Reproduced with permission from ADQI

EHR data, which are generated through the routine provision of clinical care, are “big” from the standpoint of volume (number of discrete data points available), velocity (rate at which new data accumulates), and variety (myriad of data elements available for interrogation) [3, 4]. These aspects, along with its singular clinical relevance, make EHR data ideal for disease prediction and risk forecasting. In particular, acute kidney injury (AKI) is a syndrome which lends itself well to predictive modeling and early risk stratification (Fig. 1). The presence of a standard, consensus definition allows accurate and efficient AKI diagnosis [5]; temporal anchoring of the AKI event creates a distinct pre-disease dataset to which high-content, high-throughput predictive techniques can be applied (Fig. 1). Additionally, although AKI has been associated with poor short and long term outcomes in both adults and children, no treatments exist to mitigate or cure AKI once it has developed [6–13]. The ability to predict AKI in hospitalized patients would provide the opportunity to modify care pathways and implement interventions. This, in turn, could prevent AKI events, thereby reducing mortality, shortening length of stay, averting the development of chronic kidney disease, and potentially creating novel quality of care indicators [13, 14]. In this manuscript, we present evidence informed, consensus driven statements regarding the concepts of primary relevance when considering the capacity of EHR data to be used in AKI prediction applications.

## Methods

This consensus meeting following the established ADQI process, as previously described [15]. The broad objective of ADQI is to provide expert-based statements and interpretation of current knowledge for use by clinicians according to professional judgment and identify evidence care gaps to establish research priorities. The 15th ADQI Consensus Conference Chairs convened a diverse panel representing relevant disciplines from five countries from North America and Europe around the theme of “Acute Kidney Injury in the Era of Big Data” for a 2-day consensus conference in Banff, Canada on September 6–8, 2015. During the pre-conference phase of the meeting, each work group performed a comprehensive literature search to summarize areas where broad consensus exists, categorize knowledge gaps, and identify future priorities for research. Specifically for the AKI prediction workgroup, the literature search was conducted using the terms “acute kidney injury prediction”, “acute renal failure prediction”, and “AKI prediction” in MEDLINE using PUBMED as the search engine. This search yielded a total of 679 articles for review. Studies were limited to articles published in 2010–2015 to reflect more recent harmonized AKI definitions. Studies were included if they discussed a prediction model and did not isolate the analysis to identification of independent risk factors. Studies were excluded if the focus of the prediction model was novel biomarkers due to practical issues in using these markers in current clinical practice. Thirty-four articles were selected in the initial review. Upon reviewing the articles, there was a consensus amongst work group members to include seven additional articles published prior to 2010; these articles used earlier consensus definitions for AKI, laid the groundwork for the subsequently developed models, and were archetype models when published [16–22]. Four core questions/concepts were crafted for presentation to the entire ADQI consensus group during the conference (Table 1). During the conference our work group developed consensus positions, and plenary sessions involving all ADQI contributors were used to present, debate, and refine these positions. Following the conference this summary report was generated, revised, and approved by all members of the work group.

**Table 1:** Core Questions for ADQI Consensus Group

Question 1	Across the spectrum of AKI, which event or events should be targeted for prediction?
Question 2	For the purposes of predictive modelling, what paradigm should be used for variable identification and selection?
Question 3	What is the optimal technical approach for model building and EHR integration?
Question 4	What is the optimal output of an architype predictive model?

## Results

### Question 1: Across the spectrum of AKI, which event or events should be targeted for prediction?

Prior to developing a model, it is important to carefully choose the target for prediction. From the outset, the consensus group believed it was imperative that, for the purposes of prediction, AKI be diagnosed and identified according to the generally accepted consensus definition and classification scheme, the KDIGO criteria [5]. This is the most current consensus definition, it harmonizes the previously proposed AKI criteria (RIFLE, pRIFLE, and AKIN), and is applicable to both adults and children [5, 23–25]. In order to build the strongest and most useful predictive model, we would recommend forecasting AKI events with a horizon of 48–72 h. While it would be advantageous to identify AKI events as early as possible, lengthening the event horizon reduces the accuracy of the model; we believe the suggested horizon gives practitioners adequate time to modify practice, optimize hemodynamics, and mitigate potential injury without sacrificing predictive power. The group additionally believed that rather than targeting all AKI, it would be initially advantageous to predict “moderate/severe” AKI as defined as KDIGO stage 2 or 3. While this recommendation is based on evidence-informed opinion, there are rational justifications for making it. First, this is consistent with the initial ADQI consensus statement which described the RIFLE criteria; operationally, Stage 1 KDIGO-defined AKI correlates with RIFLE stage “Risk” [24]. Treating KDIGO-defined Stage 1 AKI as “AKI risk,” allows it to become a subsequent predictor for moderate/severe AKI. Second, AKI predictors or risk factors have traditionally been more strongly associated with higher severity AKI [26, 27]. The greater strength of association will likely result in more powerful predictive modeling by reducing confounding; the development of robust models is of paramount importance for these initial big data attempts at predictive AKI analytics. Finally, while “mild” Stage 1 AKI has been associated with poorer outcomes, the association with these outcomes is significantly stronger for Stages 2/3 [6, 11, 27–31]. This ability to strongly link AKI with outcomes has an additional benefit as it will allow the models to predict not only AKI, but AKI-related outcomes as well. In one potential scenario proposed by the workgroup, a model would provide predictive AKI risk up until the occurrence of AKI then, at the inflection point of AKI development, it would provide a one-time predictive risk for patient-centered, clinically important outcomes. The workgroup acknowledges that if only Stage 2 and 3 AKI are targeted for prediction, early simulative subanalysis should be performed to evaluate the suitability of this approach.

#### Consensus Statement

For the purpose of developing AKI prediction models using the data contained within the EHR, the prototype should predict risk both for developing KDIGO-defined Stage 2/3 AKI as well as patient-centered and clinically important AKI-related outcomes.

### Question 2: For the purposes of predictive modelling, what paradigm should be used for variable identification and selection?

Prior to applying “big data” analytics to AKI prediction, the consensus group believed it was important to appraise the AKI prediction models which had been developed to date. Based upon our predictive goals outlined in the prior section, model variables of particular interest would be causally and/or temporally associated both with the development of AKI and with AKI-related outcomes.

A number of investigators have approached AKI prediction using standard multivariable regression methodology [17–22, 32, 33]. Models have been developed for a variety of patient populations with a particular emphasis on cardiac surgery patients [34, 35]; notably, less work has been performed in general critical care populations despite the fact that they are also at high risk for AKI [36–38]. Even less established are prediction models in non-critically ill patients. However, given the ultimate goal of preventing AKI, we also need to consider predictive modeling in these populations in order to identify high-risk patients as early as possible [39, 40]. A fairly comprehensive list of studies and variables are shown in Table 2. Variables from patient-specific models are often constrained to the clinical care specific to that population; for example, models for cardiac surgery patients include cardiopulmonary bypass time and number of bypass grafts. However, a number of variables commonly appear across many of the existing models (i.e., age, baseline renal function, medications, diabetes, hypertension, etc.); these variables may be better suited for a generalized model. Most models had modest predictive success with area under the receiver operating curves (AUC) approximating 0.75; a few models reached AUCs as high as 0.9, although the sample sizes were smaller and there was a pre-selection of high-risk patients [41–44]. Regardless of their ultimate utility in defining predictive variables, these models give us a minimum AUC threshold to target for successful model development.

**Table 2:** Selected list of Predictive Models Currently Available in the Literature

Study	Population and sample Size	Variables in Model	Outcome
Aronson A et al, 2007 [91]	CABG patients *n* = 2381	Age, preoperative CHF, prior MI, preexisting renal disease, intraoperative inotropes, intraoperative intra-aortic balloon pump, bypass time, pre-operative pulse pressure.	postoperative creatinine ≥2 mg/dL w/ increase ≥0.7 mg/dL from baseline or dialysis
Basu RK et al. 2014 [29]	Pediatric critically ill patients *n* = 584 total in 4 cohorts	Vasopressor/inotrope use, invasive mechanical ventilation, percent fluid overload, change in creatinine clearance	KDIGO Stage 2/3 AKI
Brown JR et al, 2007 [92]	CABG patients *n* = 8363	Age, female, diabetes, peripheral vascular disease, CHF, hypertension, prior CABG, preoperative IABP, elevated WBC count	eGFR <30 ml/min/1.73 m^2^
Chawla LS et al, 2013 [37] Koyner JL et al, 2015 [93]	Critically ill patients *n* = 77	Urine output measurement	Progression to AKIN stage III
Chong E et al, 2012 [94]	Patients w/ eGFR <60 ml/min/1.73 m^2^ undergoing percutaneous coronary intervention *n* = 770	Age, baseline eGFR, post-percutaneous coronary intervention, creatinine kinase, contrast volume	Contrast-induced nephropathy defined as 25 % or 0.5 mg/dL increase from baseline creatinine within 48 h after PCI
Cruz DN et al. 2014 [95]	Critically ill patients *n* = 506	Age, diabetes, cardiovascular disease, chronic kidney disease, hypertension, obesity, hyperbilirubinemia, cerebrovascular accident, AIDS, cancer, hypotension, high-risk surgery, nephrotoxin exposure, sepsis	AKI stage II and III defined by AKIN
Demirjian S et al, 2012 [44]	Cardiac surgery patients *n* = 25,898	gender, race, weight, pulmonary disease, CHF, diabetes, hypertension, type of surgery, previous cardiac surgery, emergency surgery, eGFR, albumin, bicarbonate, sodium, BUN, hemoglobin, platelet count, bilirubin, BMI, potassium, CPB time, intrasurgical transfusion or vasopressor, intrasurgical UOP	AKI requiring dialysis
Forni LG et al. 2013 [39]	Patients admitted to an acute medical unit *n* = 3707	Age, alertness scale, chronic kidney disease, congestive cardiac failure, diabetes, liver disease	AKI defined per KDIGO guidelines
Gao et al. 2014 [96]	Coronary angiography intervention *n* = 3945	Age, hypertension, acute MI, heart failure, use of intra-aortic balloon pump, decreased glomerular filtration rate, contrast volume	Increase in serum creatinine level
Grimm JC et al 2015 [97]	Lung transplant *n* = 10,693	Race, sarcoidosis, diabetes, weight, baseline renal function, Kanofsky performance score, previous ICU stay, ECMO, days on list, double transplant	AKI requiring dialysis
Gurm HS et al. 2013 [98]	Patients undergoing a percutaneous coronary intervention *n* = 68,753	age, weight, height, percutaneous coronary intervention status and indication, coronary artery disease presentation, cardiogenic shock, heart failure, ejection fraction, diabetes, CKMB, creatinine, hemoglobin, troponin I and T	≥0.5 mg/dl increase in serum creatinine level from baseline, RRT receipt
Ho J et al, 2012 [99]	Cardiac surgery patients *n* = 350	Bypass time, baseline eGFR, euroSCORE, postoperative serum creatinine	AKI by AKIN criteria
Hong SH et al. 2012 [100]	Living donor transplant recipients *n* = 429	age, MELD, hypertension, platelet count, surgical time, packed red blood cell transfusion, lactate, furosemide dose, calcium chloride dose, phosphate level	Renal failure was defined according to RIFLE
Kane-Gill et al. 2015 [38]	Elderly, critically ill *n* = 25,230	age, gender, race, eGFR, heart failure, diabetes, hypertension, admission type (medical vs surgical), requirement for vasopressors or mechanical ventilation, sepsis, hypotension, nephrotoxic drugs.	AKI by KDIGO criteria
Kim JM et al. 2014 [46]	Liver transplant recipients *n* = 153	Hepatic encephalopathy, deceased donor liver donations, MELD score, intraoperative blood loss, and indication for liver transplantation	Patients who needed renal replacement therapy
Kim MY et al, 2011 [101]	Isolated off-pump CABG patients *n* = 448	High systolic blood pressure, low baseline eGFR, coronary angiography less than 7 days prior to surgery	AKI by AKIN criteria
Kim WH et al. 2013 [102]	Aortic surgery with cardiopulmonary bypass *n* = 737	Age, preoperative glomerular filtration, ejection fraction, operation time, intraoperative urine output, intraoperative furosemide use	AKI defined by RIFLE
Kristovic D et al. 2015 [26]	Cardiac surgery patients *n* = 1056	age, atrial fibrillation, CHF classification, previous cardiac surgery, creatinine, endocarditis, weight, gender, COPD, bypass	AKI stage by KDIGO AKI requiring dialysis
Legrand M et al. 2013 [103]	Patients with endocarditis/ cardiac surgery with cardiopulmonary bypass *n* = 202	Age, gender, pre-existing comorbidities, presence of shock, systemic emboli, NYHA classification, hemoglobin, baseline creatinine, need for mechanical ventilation, characteristics of infection/surgery, use of nephrotoxic agents	Development or progression of AKI in the 7 days following surgery. AKI defined per AKIN
McMahon GM et al. 2013 [104]	Rhabdomyolysis within 3 days of admission *n* = 2371	Age, female sex, cause of rhabdomyolysis, initial creatinine, creatinine phosphokinase, phosphate, calcium, and bicarbonate	Composite endpoint: Renal replacement therapy or mortality
Medha et al. 2013 [41]	Trauma patients *n* = 4396	hepatic dysfunction, urea, glucose, pulmonary dysfunction, severity of injury	serum creatinine level >2.0 mg/dL during the hospital stay
Meersch M et al. 2014 [42]	Patients undergoing cardiac surgery with bypass *n* = 50	Diabetes, severity of illness, ejection fraction, baseline serum creatinine, cross-clamp time, chronic obstructive pulmonary disease	AKI defined by RIFLE or AKIN together
Mehran R et al, 2004 [22]	Patients undergoing percutaneous coronary interventions *n* = 8357	Hypotension, intra-aortic balloon pump, congestive heart failure, age >75 years, anemia, diabetes, contrast volume, baseline creatinine or eGFR	Increase of ≥25 % or ≥0.5 mg/dL in pre-PCI serum creatinine at 48 h after PCI
Mehta RH et al, 2006 [32]	CABG and/or valve surgery patients *n* = 449,524	Preoperative creatinine, age, race, type of surgery, diabetes, shock, NYHA class, lung disease, recent myocardial infarction, prior cardiovascular surgery	AKI requiring dialysis
Ng SY et al. 2014 [105]	Cardiac surgery patients *n* = 28,422	obesity, infective endocarditis, cardiac procedure, preop creatinine, diabetes, urgency status, eGFR, CHF, age, cardiogenic shock, IABP use, bypass time, non-RBC blood product use, gender, reoperation for bleeding, hypercholesterolemia, hypertension, and respiratory disease	Increased creatinine > 200 mmol/L (2.26 mg/dL), ≥ 2x increase in creatinine over baseline, a new receipt for RRT
Palomba H et al. 2007 [18]	Cardiac surgery patients *n* = 603	age, serum creatinine, glucose, heart failure, combined surgeries, cardiopulmonary bypass time, cardiac output, central venous pressure	creatinine > 2.0 mg/dl or increase of 50 % above baseline
Park MH et al. 2015 [106]	Living-donor liver transplant *n* = 538	weight; diabetes, alcoholic liver disease, albumin <3.5 mg/dL, model for end-stage liver disease score, child-turcotte-pugh- estimated graft to recipient body weight ratio, operation details, calcineurin inhibitor use without mycophenolate	AKI as defined by RIFLE
Rahmanian PB et al, 2011 [107]	Cardiac surgery patients *n* = 2511	Pulmonary hypertension, preoperative renal dysfunction, bypass time, peripheral vascular disease, recent MI, atrial fibrillation, age, CHF, diabetes	AKI requiring dialysis
Rodriguez et al. 2013 [108]	Severe Rhabdomyolysis *n* = 126	Albumin, metabolic acidosis, prothrombin time, peak creatinine phosphokinase	RIFLE category
Romano TG et al. 2013 [109]	Orthotopic liver transplant patients *n* = 114	MELD	increase ≥ 0.3 mg/dL in serum creatinine
Schneider DF et al, 2012 [110]	Critically ill burn patients *n* = 309	age, sex, race, % body surface area burned, burn mechanism, intubation, inhalation injury, NROF, fraction of predicted Parkland resuscitation, early transfusion, weight, Charlson Score, drug abuse, smoker, number of preadmission medications, ACEI/ARB, diuretic, NSAIDs, methamphetamine, lowest hematocrit, potassium, sodium, pH, glucose, base deficit, lowest mean arterial pressure, temperature	AKI defined using RIFLE classification
Simonini M et al. 2014 [111]	Elective cardiac surgery *n* = 802	Age, gender, ejection fraction, hypertension, diabetes, renal function, reoperation cardiac surgery, surgery type	AKIN stage II/III AKI
Slankamenac K et al. 2013 [112]	Liver surgery *n* = 549	Need for blood transfusion, cirrhosis, oliguria, hepaticojejunostomy, use of colloids, use of diuretics, use of a bolus of catecholamines	R of RIFLE
Soto K et al. 2013 [40]	Patients admitted from the emergency department *n* = 616	Age, kidney susceptibility stage, chronic heart failure, hypertension, cardiovascular disease, and diabetes mellitus	New onset AKI per RIFLE
Thakar CV et al, 2005 [17]	Cardiac surgery patients *n* = 31,677	gender, CHF, ejection fraction, preop intra-aortic balloon pump, COPD, diabetes, previous cardiac surgery, emergency surgery, type of surgery, creatinine >1.2	AKI requiring dialysis
Tsai TT et al. 2014 [113]	Percutaneous coronary intervention *n* = 947,012	Age, CKD, prior cardiovascular disease, acute coronary syndrome, cardiac arrest, anemia, CHF, intra-aortic balloon pump prior to procedure, cardiogenic shock	AKI defined by AKIN and AKI requiring dialysis
Wang M et al. 2013 [114]	Patients with hemorrhagic fever (Hantann virus) *n* = 112	age, gender, presence of shock, proteinuria, hematuria, platelet count, leukocyte	Required dialysis or increased serum creatinine ≥354 mmol/L
Wang Y et al. 2013 [115]	Patients hospitalized with acute heart failure *n* = 1709	Age, ≥ 3 previous hospital admissions for acute heart failure, systolic blood pressure <90 mmHg, serum sodium <130 mmol/L, heart functional class IV, proteinuria, SCr ≥ 104 mmol/L, intravenous furosemide dose ≥ 80 mg/day	increase in serum creatinine (SCr) of ≥26.4 mmol/L or ≥ 50 % within 48 h.
Wijeysundera DN et al, 2007 [19]	Cardiac surgery patients *n* = 20,131	Preoperative eGFR, diabetes, ejection fraction, previous cardiac surgery, procedures other than isolated CABG or ASD repair, non-elective procedure, preoperative intra-aortic balloon pump	AKI requiring dialysis
Wong B et al. 2015 [116]	Cardiac surgery patients who developed AKI *n* = 2316	Age, weight, preoperative creatinine, gender, preoperative intra-aortic balloon pump, ejection fraction, type of surgery, previous cardiac surgery, diabetes, COPD, cardiopulmonary bypass time, clamp time, pump time, number of bypass grafts	AKI Stage 1, stage 2, stage 3
Xu X et al, 2010 [117]	Liver transplant recipients *n* = 102	age, MELD score, preoperative creatinine, BUN, sodium, and potassium, intraoperative UOP, intraoperative hypotension, intraoperative noradrenaline	Serum creatinine >1.5 mg/dl with an increase of 50 % above baseline and/or RRT

As stated, ideal variables would be associated with both the development of AKI and patient centered, clinically important outcomes following AKI. Notably, many of the same risk factors described in Table 2 as predicting AKI occurrence have also been shown to predict AKI-associated mortality [36, 45–51]. In addition to these factors, positive fluid balance has been associated with increased mortality in both pediatric and adult patients with AKI [52–56]. Receipt of renal replacement therapy (RRT) is another outcome worth forecasting after AKI has occurred. Although most of the published clinical scores predicting receipt of RRT have focused on post-cardiac surgery patients, they have identified many of the same predictors for AKI occurrence in broader populations [17, 19, 32, 34]. AKI is known to be associated with the development of CKD and ESRD, therefore, prediction of these long-term outcomes among AKI survivors should also be targeted; archetype variables associated with these outcomes are shown in Table 2 [8, 57–68].

**Fig. 2:**
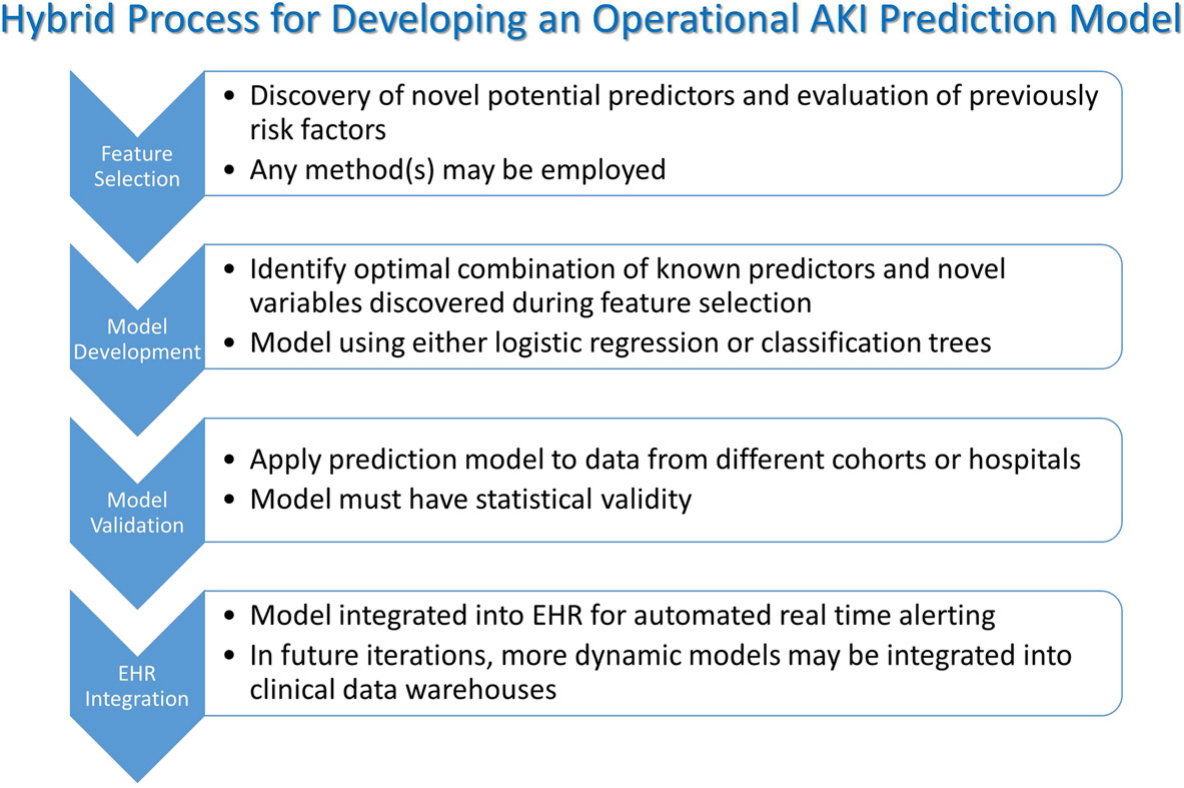
Development of AKI Prediction Algorithm. The first step in the development of an AKI prediction model is feature selection. This process would evaluate known risk factors identified from the literature and would use machine learning techniques to identify novel risk factors from amongst the EHR dataset. All appropriate features would be considered for inclusion in the actual prediction model which would weight individual variables to create a generalizable model. This model would be validated using a different (or subset of existing) dataset. Once validated, the model could then be integrated directly into the EHR to allow real time AKI alerting. Reproduced with permission from ADQI

While the group believed it was imperative that previously identified AKI predictors be reviewed, to truly harness the power of the EHR a de novo approach which considers the entirety of the dataset is required (Fig. 2). There are a number of potential data-mining, machine learning approaches to this sort of feature selection which could be used alone or in combination (Table 3). One method, neural networks, employ non-linear models that feed a set of predictors (inputs) into a hidden layer of units which then use a non-linear transformation to send a value to an output; the prediction is a summation of all the values coming from the hidden layer [69]. This non-linear technique is sometimes described as a “black box” method since it’s difficult to determine the form of the final predictive model. However, this is of little concern in feature selection, as identifying the group of the most influential variables is of interest. A second potential method is that of random forests which is an extension of the binary split classification tree approach [70, 71]. Multiple trees are created by allowing a random number of the predictor variables to be considered at each split of each tree. This results in trees that cover a larger solution space, potentially increasing accuracy. Another set of methods is cluster analysis, a group of unsupervised learning techniques. Observations are grouped according to their similarities in a multidimensional space, based on a distance measure [72]. The resultant clusters can then be further explored to see which ones have a very high or a very low incidence of the outcome measure. A similar method is a technique known as self-organizing maps, in which unsupervised neural networks map a highly dimensional space onto a two-dimensional map [73, 74]. Principal components analysis and support vector machines are two similar feature selection methods which could be employed in this space [75, 76]. Although this is not a comprehensive list of the methods that might be considered, these are excellent exemplar techniques which can be used to identifying novel features and transform known risk factors.

**Table 3:** Big data modeling techniques

Method	Advantages	Disadvantages
Neural Networks	Discover non-linear relationships. Can assess multi-level interactions	“Black Box” to clinicians; hard to implement into a DSS*
Random Forests	Finds most probable solution set; robust against scaling influences	Not always best in terms of prediction; hard to implement into a DSS
Cluster Analysis	Finds groups of very similar patients; exploratory analysis	Unsupervised technique
Principal Components Analysis	Uncovers the variables contributing the most to outcome variation	Not amenable to binary outcomes; assumes additive relationship
Support Vector Machines	Robust against statistical assumptions	Difficult to implement into a DSS

In summary, the suggested approach highlights our belief that accurate prediction of AKI takes precedence over finding putative variables, though the suggested approaches do not preclude discovery of new risk factors for AKI. Additionally, while it is useful to review previously established variables associated with AKI from existing studies, application of high content, machine learning techniques to the complete EHR dataset will be the driving force behind variable selection. The ability to dynamically identify and integrate variables from amongst innumerable patient-level data elements represents a marked departure from classically developed model building approaches.

#### Consensus Statement

Variables included in prototype AKI prediction models should be identified using a hybrid approach; risk factors which are well established in the literature should be considered along with novel risk factors identified via machine learning techniques. Application of these unsupervised approaches should take precedence as it allows feature selection to be dynamic, thereby generating the strongest prediction from existing data elements.

### Question 3: What is the optimal approach for model building and EHR integration?

Once the aforementioned hybrid variable selection process was complete, previously identified risk factors and potential predictors discovered via big data techniques could be considered for inclusion in a model. Inclusion criteria could include:

1. Evidence over multiple studies that the risk factor was a powerful predictor of AKI
2. Identification by machine learning techniques to be predictive of AKI and outcomes
3. Available discretely within the EHR to allow easy integration
4. Reliably/accurately recorded within the EHR

Variables need not necessarily be universal. For example, pediatric or ICU specific variables could be considered; the model could be dynamic with certain features active/inactive in certain locations/populations. Additionally, it is possible that effect modification of the variables could vary between patients or populations; the presence or absence of certain variables might change the weighting of the residual variables.

While we advocate for a big data approach to identify novel predictive features, initially we would recommend that the predictive model itself be built through more standard statistical modelling. This is primarily due to the inherent limitations of current EHR architecture. EHRs are built to optimize patient level data review and display; they are not necessarily organized to optimize cohort level analysis [77]. This makes implementation of a resource-intense machine learning algorithm into the EHR itself technically and operationally problematic. Therefore, once the variables were identified by literature search and machine learning methodology, it is likely that a logistic regression model, discriminant analysis, or decision tree algorithm would be employed to predict the development of AKI [71, 78, 79]. Data could accumulate on a “rolling window” concept and a prediction could be generated at a pre-specified interval (hourly, every two hours, every shift); alternatively, the model could generate a score in real time as each new data value is received. One conceptual approach would allow this model to generate a risk score ranging from 0 to 100; low scores would be indicative of minimal AKI risk and high scores would be indicative of significant AKI risk. Scoring on a continuous scale would allow both low and high thresholds to be set. In many ways, the ability to identify patients at negligible AKI risk could be as valuable as identifying patients at great AKI risk. An algorithm such as this could be active up until the time the patient develops AKI. At that inflection point, a final, one-time score could be generated which would be reflective of the patients AKI-related outcome risk, thereby allowing practitioners to identify patients at great risk for poorer outcomes.

It is important to note that while the EHR has operational and structural limitations to the application of big data techniques, alternatives should be available in the future. For example, many clinical data warehouse (CDW) solutions have become available for analytic purposes [80–83]. These CDWs represent “shadow” EHRs in which data has been manipulated, linked, and stored in a fashion conducive to high-content, high-throughput analytics [82, 83]. Once such CDWs become as ubiquitous as EHRs, big data approaches could be applied directly to the CDW environment. However, to truly exploit the full capacity of the EHR and EHR data, a more progressive approach is necessary. The EHR has transcended its original purpose; although it is currently a care monitoring and delivery tool, it has the potential to revolutionize clinical care paradigms. To achieve this, data architecture must become as important as data entry and analytics must be prioritized. The creation of a true “learning EHR” could be the key to higher quality, lower cost care delivered with greater efficacy and efficiency.

#### Consensus Statement

While machine learning techniques should be used to identify novel AKI risk factors, prototype AKI prediction models should be built using more standard statistical weighing techniques to allow effective EHR integration. However, analytics should attain higher priority and the operational limitations of the EHR should be addressed. Consequently, subsequent predictive iterations should progress towards full EHR-integration of high content analytic techniques.

### Question 4: What is the optimal output of an archetype predictive model?

**Fig. 3:**
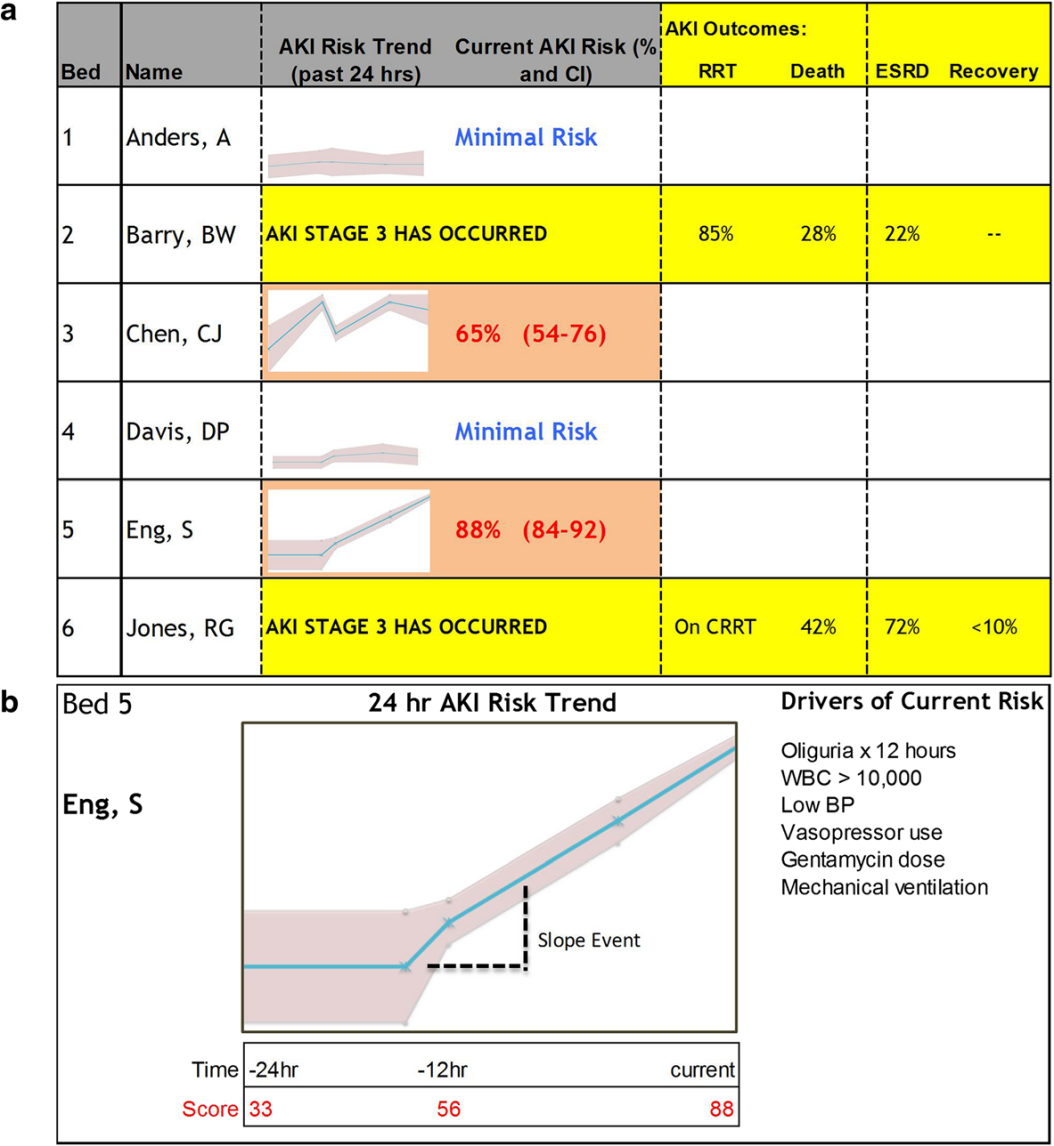
**a** and **b** Renal Dashboard. Once the risk prediction model is developed and validated, it is important to determine how to deliver the information to providers. One possible output might be a “Renal Dashboard” (**a**). The display would visually display the time trend of AKI as well as a numeric value (with confidence intervals) for the current risk. For any patients who develop AKI, information about outcome risk would be provided; in this example, the outcomes of interest are need for RRT, mortality, development of ESRD, and likelihood of renal recovery. The dashboard could be dynamic, allowing providers to drill into the risk score. In the patient level display (**b**), information would be available about how the risk had trended over the past 24 h as well as what factors were affecting the current risk score most significantly. In this example, AKI risk information is provided in a visually stimulating manner with a dynamic component capable of driving care modification. Reproduced with permission from ADQI

After the rigorous steps undertaken to select variables and develop a predictive model, we propose that any prototypes be directly integrated into the EHR for automated real time usage. The increasingly widespread use of EHRs across hospitals has substantially increased the amount of data available to providers [84]. However, while EHRs purportedly improve patient outcomes, studies that have validated these benefits are lacking [85–87]. Several potential EHR-related barriers to improving outcomes have been identified and include information overload, ineffective data display, and poor implementation processes [88–90] Therefore, it is imperative that an AKI prediction model not only harness the power of the EHR data set, but also that it effectively conform to the strengths and limitations of EHR processes. Ideally, AKI risk prediction tools should directly extract relevant data predictors in real-time, deliver a relevant “renal risk score,” and provide feedback to practitioners regarding potential actionable items. One potential a concept would be to create a “renal dashboard” (Fig. 3a and b).

The main objective of the renal dashboard would be to provide feedback on the absolute risk of developing moderate to severe AKI within the next 48–72 h as well as to present information about the clinical features contributing to these risks. The electronic dashboard format could be tailored for a particular provider, service, or unit. Each patient could have a risk score (in percentage) with an accompanying confidence interval (Fig. 3a); a confidence interval component would give practitioners an idea of how certain the AKI risk was at any given time. In addition to absolute risk scores, the dashboard could be configured to display time trends in risk scores which might give a better sense of evolving AKI risk. Time trends should be displayed in a visually stimulating fashion (i.e., sparklines) to demonstrate the dynamic nature of real-time AKI-risk. A fully optimized dashboard might allow providers to “drill into” the risk score (Fig. 3b), revealing a magnified view as well as more detailed data on the most recent predictors that contributed to a significant increase in risk score. The identification of specific vital sign indicators, laboratory parameters, medication administration data, or other clinical factors that contributed directly to a rise in AKI risk will help guide providers toward implementing risk reduction actions.

A secondary objective of the dashboard might be to provide updated feedback on the risk of adverse outcomes associated with AKI once it actually develops. Early iterations of this sort of prototype may be limited to one-time scores for AKI-related outcomes. However, at the inflection of AKI development, separate risk scores for mortaltiy, receipt of RRT, CKD, and renal recovery could be provided. As an example, the ability to predict receipt of RRT may help providers plan for appropriate patient disposition (i.e., transfer to ICU for CRRT) and timely procedures (i.e., placement of dialysis catheter). Prediction of long-term renal and cardiovascular outcomes could be especially useful at the time of discharge, facilitating appropriate referrals, vascular access planning, and long-term care goal discussions.

We anticipate that a renal dashboard such as this could be displayed either directly within the system or independently from the EHR platform. Although information would be directly fed to the prediction model from up-to-date EHR data, each healthcare system, service, or unit may tailor the physical setting of the dashboard display to fit their workflows. For example, in an ICU setting where incidence of AKI may be as high as 40 %, the renal dashboard may be displayed on computerized workstations on wheels so that providers can incorporate the real-time information and feedback provided by the renal dashboard into their multi-disciplinary rounds [31]. For other services and locations where incidence of AKI is much lower - for example, the labor and delivery unit - the renal dashboard may serve in a more adjunctive role, to be monitored by a specialized “renal response” team (akin to traditional “rapid response” teams).

The consensus group acknowledges that numerous such dashboards could be created for similar medical conditions to assist with risk stratification. The approach described in this manuscript is designed to underscore the utility of a dashboard scheme. We realize that developing multiple dashboards for individualized diseases is unlikely to be efficient or effective in the long run. Operationally, a superior approach would be to seamlessly integrate a renal dashboard component into existing dashboard which is used to evaluate a range of quality and performance indicators.

#### Consensus Statement

The output from predictive models should be delivered to practitioners in a fashion that is cognizant of EHR limitations and strengths, minimizes workflow inefficiency, and maximizes utility.

## Conclusion

The EHR dataset is a massive collection of clinically relevant data elements generated through the routine provision of patient care. Its size and complexity lend themselves to “big data” techniques; these in turn offer the potential to use the entire EHR dataset to predict AKI and AKI related outcomes. Variable selection should employ high-content, unsupervised analytic techniques. Developing predictive models should focus on EHR integration and optimize the output for clinical utility.

## Abbreviations

AKI: acute kidney injury
KDIGO: Kidney Disease: Improving Global Outcomes
RIFLE: Risk, Injury, Failure, Loss, ESRD
AKIN: Acute kidney injury network
ADQI: Acute Dialysis Quality Initiative
EHR: Electronic health record
